# Spurious or Island Effect? A Response to J. A. Pérez-Claros and J. C. Aledo's Comment on “Morphological Evolution Is Accelerated among Island Mammals”

**DOI:** 10.1371/journal.pbio.0050176

**Published:** 2007-07-17

**Authors:** Virginie Millien

In their comment [1], Pérez-Claros and Aledo claimed that the main result of accelerated evolution in island mammals, compared with their mainland relatives, is not well supported by the data and analyses presented in [2]. They raise two points: the first refers to a long-standing methodological debate regarding the study of evolutionary rates and the second refers to the quality of the dataset in [2].

Haldane [3] first proposed the unit darwin to calculate rates of morphological evolution in fossil taxa. Since then, there has been much controversy on the relevance of the darwin as a measure of evolutionary rates [4].

First, evolutionary rates in darwins that are calculated over the largest time intervals are necessarily lowered, because they average periods of rapid change with periods of slow change or stasis, and they can also include fluctuations in the direction of change [4]. Second, because evolutionary rates are dependent on the time interval over which they are measured, rates measured over different time intervals cannot be compared without taking into account the time interval over which they were calculated (i.e., temporal scaling), which is often done by plotting rates against time intervals on a log-log graph. This is equivalent to plotting a ratio (rate) against its denominator (time interval), which produces a negative correlation and may be a mathematical artifact that has been dubbed “the spurious effect” [5,6].

Despite these two important caveats, rates in darwins are still useful to compare similar or different lineages evolving at similar or different times and places [7].

More importantly, these concerns simply do not affect the results presented in [2], because the conclusions were solely based on the comparison of the elevation of the two regression lines. The extent to which the “spurious effect” influences the slopes of the regression is not of direct relevance to the question of whether mammals evolve faster on islands [2]. In fact, there is no reason to believe that such a bias will introduce a systematic difference among the mainland and island groups compared in [2]. Given this, it is unlikely that the “spurious effect” can be evoked to explain the differences in the island–mainland comparison.

Another more-specific point raised by Pérez-Claros and Aledo concerns the data that was assembled to test the theory and upon which the conclusions were based in [2]. Pérez-Claros and Aledo indicate that the analysis is no longer significant when re-performed on a subset of the data. This is true, but it is easy to reduce the range of data to a point where the relation is no longer significant. Is it relevant to test a theory on a subset of data that represents less than half of the data and only 16% on a log scale (or 0.15% on a natural scale) of the original range of time intervals? I contend that their analysis reduces the dataset to such an extent that it is no longer meaningful to address the evolutionary question at hand. The data made available in [2] represent, to date, the largest dataset ever assembled on morphological rates of evolution in mammal species, fossil and recent. Yet, the data are unbalanced, with an over-representation of island taxa for the smallest time intervals, and an over-representation of mainland data for the largest time intervals. This issue was already addressed [2, page 0002], and readers are referred to the original paper for more details. In the future, as more data become available, the conclusion in [2] may be either validated or challenged, but for the time being, the difference in elevation between the two regression lines is statistically significant and is the most robust for time intervals below 20,000 y. Morphological evolution is faster in island mammals over these time intervals, when compared with that of mammals evolving on the mainland.
